# Escape Path Planning for Unmanned Surface Vehicle Based on Blind Navigation Rapidly Exploring Random Tree* Fusion Algorithm

**DOI:** 10.3390/s24237596

**Published:** 2024-11-28

**Authors:** Bo Zhang, Shanlong Lu, Qing Li, Peng Du, Kaixin Hu

**Affiliations:** 1 School of Automation, Beijing Information Science and Technology University, Beijing 100192, China; 2022020457@bistu.edu.cn (B.Z.); liqing@bistu.edu.cn (Q.L.);; 2 International Research Center of Big Data for Sustainable Development Goals, Beijing 100094, China; 3 Aerospace Information Research Institute, Chinese Academy of Sciences, Beijing 100094, China; 4 Key Laboratory of Modern Measurement & Control Technology, Ministry of Education, Beijing 100101, China

**Keywords:** Blind Navigation Rapidly Exploring Random Tree* (BN-RRT*), Rapidly Exploring Random Trees (RRT*), Artificial Potential Field (APF), escape strategy, path planning

## Abstract

To address the design and application requirements for USVs (Unmanned Surface Vehicles) to autonomously escape from constrained environments using a minimal number of sensors, we propose a path planning algorithm based on the RRT* (Rapidly Exploring Random Tree*) method, referred to as BN-RRT* (Blind Navigation Rapidly Exploring Random Tree*). This algorithm utilizes the positioning information provided by the GPS onboard the USV and combines collision detection data from collision sensors to navigate out of the trapped space. To mitigate the inherent randomness of the RRT* algorithm, we integrate the Artificial Potential Field (APF) method to enhance directional guidance during the sampling process. Additionally, inspired by blind navigation principles, we propose an active collision mechanism that relies on continuous collisions to identify obstacles and adjust the next movement direction, thereby improving the efficiency of escape path planning. We also implement an obstacle memory mechanism to prevent exploration into erroneous areas during sampling, significantly increasing the success rate of escape and reducing the path length. We validate the proposed algorithm in a dedicated MATLAB environment, comparing its performance with existing RRT, RRT*, and APF-RRT* algorithms. Experimental results indicate that the improved algorithm achieves significant enhancements in both planning speed and path length compared to the other methods.

## 1. Introduction

In recent years, unmanned detection technology has advanced rapidly, and it has been widely applied in various fields, such as land and resource surveys, ecological environmental protection, and emergency monitoring [1,2,3,4]. Water depth data are essential for assessing the total volume of water resources stored within a body of water [5]. Currently, most USVs (Unmanned Surface Vehicles) depend on sensors, such as radar and cameras, to gather external information for obstacle avoidance. The robust obstacle avoidance capability comes at the cost of being expensive, bulky, and heavy. For these USVs, acquiring large-scale water depth data requires considerable financial resources and time [6,7].

To address these challenges, our research team has integrated USV technology with water depth detection systems to propose an autonomous drifting USV designed for large water bodies, such as lakes and reservoirs (during normal operation, it relies on water flow for propulsion. When obstructed by obstacles and stationary for an extended period, the escape algorithm is activated to facilitate its release). To meet the requirements of long-term operation, cost reduction, and lightweight design, USVs are equipped with GPS [8] and collision sensing sensors [9], in addition to water depth measurement equipment. These sensors are utilized to collect accurate positional data for depth measurement points and to monitor collisions, thereby aiding in escape path planning. Given the unpredictable nature of the aquatic environment, there is a risk that the USV might become entrapped near obstacles during its drift, preventing it from continuing its mission. Therefore, developing an algorithm that leverages onboard positional information and collision awareness to enable autonomous navigation away from obstacles in unknown environments is of paramount importance.

Currently, among the mainstream path planning algorithms, those widely recognized include the A* algorithm, the Artificial Potential Field (APF) algorithm, the Rapidly Exploring Random Tree (RRT) algorithm, the simulated annealing algorithm, and the genetic algorithm. Grid-based search algorithms, typified by the A* algorithm, generally guarantee the optimality of the planned path; however, their reliance on pre-existing grid information results in suboptimal performance in unknown environments [10,11,12]. The APF algorithm boasts a straightforward structure and robust real-time capabilities, yet it suffers from issues related to local minima and path oscillation [13,14,15]. The simulated annealing algorithm avoids the problem of local minima but exhibits slow convergence [16,17,18]. The genetic algorithm can identify the optimal path, albeit at the cost of multiple iterations, which introduces complexity and diminishes real-time performance—features that render it less suitable for the application of USVs discussed herein [19,20,21]. In light of these considerations, the RRT algorithm employs a global random sampling strategy, offering the benefits of minimal parameter tuning and a robust capability to handle complex constraints. These characteristics make it particularly suitable for planning escape paths in unknown, confined environments [22,23,24]. However, the inherent randomness of the RRT algorithm can lead to instability in path planning, indicating a need for enhancements to improve its reliability.

Many researchers have improved the RRT algorithm to address its shortcomings. The RRT* algorithm, for instance, introduces a step where a circle is drawn around the new path point during the RRT process, searching within this circle for a better parent node to optimize the path, thereby making the planned path more optimal [25]. Brunner et al. (2013) utilized bias sampling to enhance the RRT* algorithm, reducing its randomness and increasing stability [26]. Nasir et al. (2013) proposed an RRT* variant that simplifies map sampling, effectively boosting the algorithm’s search efficiency [27]. Jeong et al. (2019) introduced the Quick RRT* algorithm, expanding the scope of the RRT* method through the reselection of parent nodes and the pruning of randomly sampled paths [28]. Liao et al. (2021) developed the F-RRT* algorithm, optimizing the path cost by generating parent nodes for random points, thus producing more effective initial solutions [29].

Although many researchers have made significant advancements in enhancing search efficiency, path performance, and reducing the randomness of the RRT* algorithm, these improved algorithms remain unsuitable for application in the USV system described in this article. The aforementioned improvements to these algorithms are primarily based on environmental perception through visual or radar sensors, coupled with methodologies for achieving obstacle avoidance. While the addition of sensors can improve navigation efficacy, it also increases the cost and power consumption of the USV. Currently, path planning algorithms are often based on known environments or rely on detecting and acquiring environmental information. However, there is relatively little research focused on path planning methods designed for unknown environments with limited detection capabilities.

This article proposes a novel fusion algorithm that utilizes the unmanned ship’s positional information and collision detection capabilities to achieve self-rescue in unknown, confined environments. The algorithm is specifically designed to meet the hardware constraints and operational requirements of autonomous unmanned ships engaged in drifting water depth measurement. Building upon the RRT* framework, it incorporates elements of the Artificial Potential Field (APF) algorithm to reduce randomness. Inspired by principles of blind pathfinding, the algorithm establishes active collision points to direct the unmanned ship in navigating trapped environments, thereby accelerating the rescue process. Additionally, an obstacle memory mechanism is implemented to associate detected obstacle points with designated obstacle zones, thus preventing the unmanned ship from revisiting these areas during its escape. Adaptive step size methods are employed to optimize the planned path. To evaluate the effectiveness of the proposed BN-RRT* algorithm, experiments were conducted to compare it against the RRT, RRT*, and APF-RRT* algorithms, demonstrating its superiority.

Each section of this paper is arranged as follows. In Section 2, we describe the mathematical model of the USV; in Section 3, we discuss the RRT, RRT*, and APF algorithms; in Section 4, we introduce the details and overall technical roadmap of BN-RRT*; in Section 5, the simulation results are presented to demonstrate the effectiveness of the BN-RRT* algorithm; and in Section 6, we present the conclusion of this article.

## 2. USV Mathematical Model

**Figure 1:**
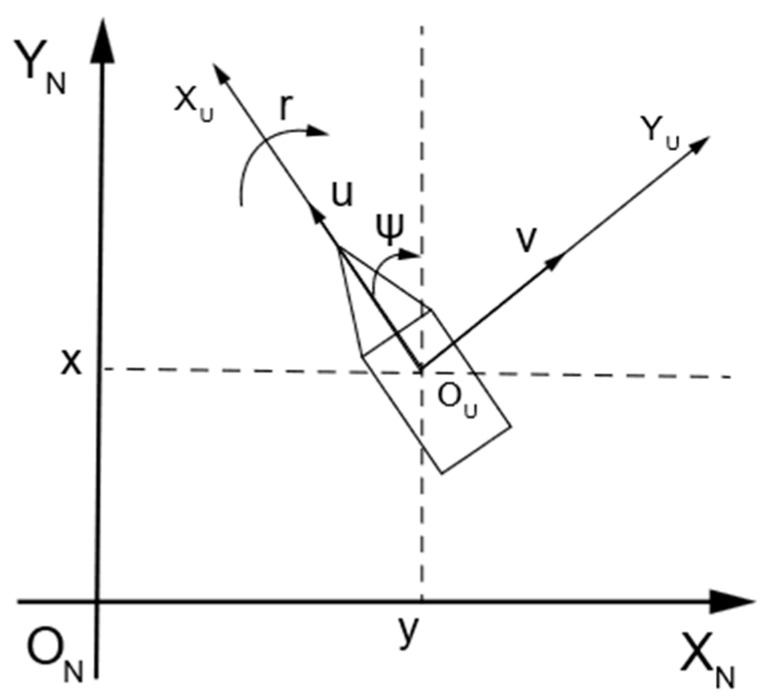
Schematic diagram of the USV mathematical model.

The mathematical model of the USV provides essential theoretical support for algorithm research and simulation experiments. When analyzing the USV’s mathematical model using coordinate systems, it is typically necessary to establish both the geodetic coordinate system and the USV body-fixed coordinate system. The geodetic coordinate system includes axes $O_{N}X_{N}Y_{N}Z_{N}$, where the origin $O_{N}$ is on the Earth’s surface, the positive $X_{N}$ axis points in the direction of true north, the positive $Y_{N}$ axis points in the direction of true east, and the positive $Z_{N}$ axis points toward the Earth’s center. The USV body-fixed coordinate system includes axes $O_{U}X_{U}Y_{U}Z_{U}$, where the origin $O_{U}$ is at the USV’s center of gravity, the positive $X_{U}$ axis points in the direction of the bow, the positive $Y_{U}$ axis points to the starboard side of the vessel, and the positive $Z_{U}$ axis points toward the center of the USV. In practical scenarios, the USV operates as a six-degrees-of-freedom (6DOF) system, capable of heaving, swaying, surging, yawing, rolling, and pitching. However, given the complexity of the 6DOF model, this paper focuses solely on the horizontal-plane motion of the USV for path planning algorithm discussion. Thus, only the surge, sway, and yaw degrees of freedom are considered. Let $\eta ={\left[x,y,\psi \right]}^{T}$ denote the position and orientation vector of the USV, and let $v={\left[u,v,r\right]}^{T}$ represent the surge, sway, and yaw vector of the USV. The horizontal motion model of the USV is illustrated in Figure 1.

## 3. Basic Principles of Algorithm

### 3.1. RRT Algorithm

**Figure 2:**
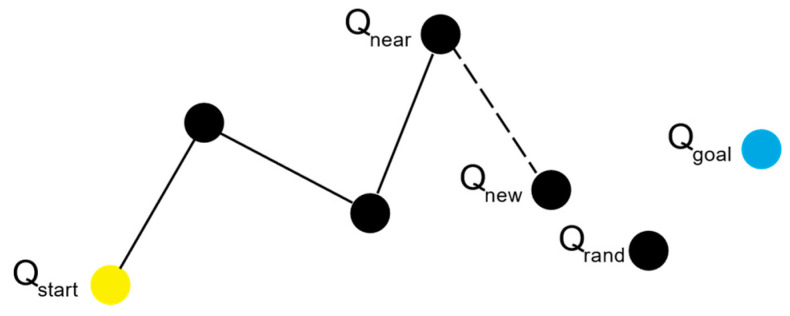
Schematic diagram of the RRT algorithm.

The RRT algorithm initializes the search tree with the starting point $Q_{start}$ of the path. Subsequently, random sampling is performed within the space to obtain a random point $Q_{rand}$. The algorithm then identifies the node $Q_{near}$ within the existing search tree that has the closest Euclidean distance to $Q_{rand}$. By extending $Q_{near}$ in the direction of $Q_{rand}$ by a fixed step size, a new node $Q_{new}$ is generated. The next step involves checking whether the line segment connecting $Q_{near}$ and $Q_{new}$ intersects with any obstacles. If no intersection occurs, $Q_{new}$ is added to the search tree. Otherwise, the node is discarded, and a new $Q_{new}$ is selected. This process is repeated, continually expanding the search tree until the distance between $Q_{new}$ and the goal point $Q_{goal}$ falls below a predefined threshold, at which point the iteration ceases. This results in a collision-free path from the starting point to the target point. Figure 2 illustrates the process of the RRT algorithm.

### 3.2. RRT* Algorithm

**Figure 3:**
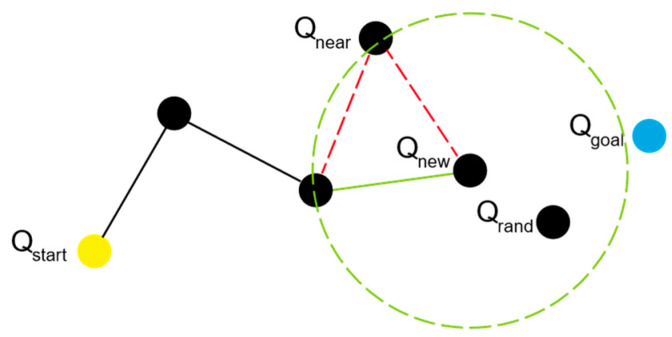
Schematic diagram of the RRT* algorithm.

The RRT* algorithm extends the RRT framework by incorporating a progressive optimization function. As the algorithm iterates, the structure of the search tree nodes is continuously refined. Compared to the RRT algorithm, the RRT* algorithm can yield shorter paths. When determining $Q_{new}$, the algorithm takes this new node as the center and sets a value as the radius to search for the shortest path to reach $Q_{new}$ within that radius. Upon identifying the path, the algorithm checks whether the connection between the nodes collides with any obstacles. If no collision is detected, the parent node is updated, and the original connection is removed. After determining the optimal parent node, the algorithm traverses the remaining nodes, recalculating the path cost for each node to reach $Q_{new}$, and it selects the shortest path to reconstruct the search tree. Figure 3 illustrates the process of the RRT* algorithm.

### 3.3. APF Algorithm

The Artificial Potential Field method for path planning is a virtual force approach proposed by Khatib. This method defines the operating environment of the intelligent agent as an abstract potential field, which is the superposition of a gravitational field directed toward the target location and a repulsive field emanating from obstacles in the environment. These two fields work in concert to guide the intelligent agent toward the target point.

**Figure 4:**
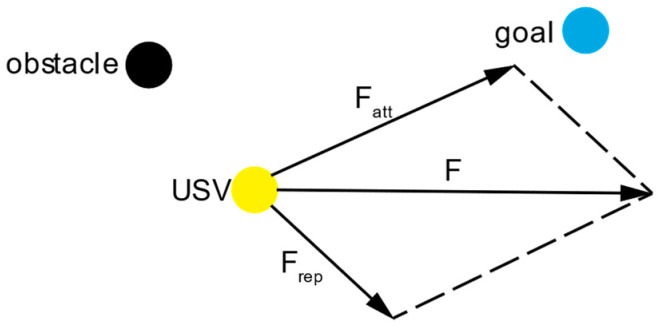
Schematic diagram of the APF algorithm.

Due to the inherent randomness in the RRT* algorithm when sampling random points, it is necessary to integrate the APF algorithm to enhance the directionality toward the target during the search process. This involves applying an attractive force from the target point and a repulsive force from the obstacle points. By overlaying these two potential fields, a resultant potential field is created, guiding the unmanned ship toward the target point. Figure 4 illustrates the process of the APF algorithm.

The attractive potential field is established around the target point and exerts a guiding influence on the USV. The farther the USV is from the target point, the greater the attractive potential energy. The function of the attractive potential field is defined as follows:

(1) $$U_{att}(X)=\frac{1}{2}k_{a}P^{2}(X,X_{g})$$

In the formula, $U_{att}$ represents the attractive potential field generated by the target point acting on the USV, $k_{a}$ is the coefficient of the attractive potential field, X is the current position of the USV, $X_{g}$ is the position of the target point, and $P\;(X,X_{g})$ denotes the Euclidean distance from the USV to the target point. The attractive force $F_{att}$ acting on the USV is derived from the negative gradient of the attractive potential field function, and the formula is

(2) $$F_{att}(X)=-\nabla U_{att}(X)=-k_{a}P(X,X_{g})$$

The repulsive potential field is established around the obstacle point and exerts a repulsive effect on the USV. The closer the USV to the obstacle, the greater the repulsive potential energy. The function of the repulsive potential field is

(3) $$U_{rep}(X)=\left\{\begin{array}{c} \frac{1}{2}k_{r}\left(\frac{1}{P(X,X_{o})}\right.-{\left.\frac{1}{d_{o}}\right)}^{2},0\leq P(X,X_{o})\leq d_{o}\\ 0,P(X,X_{o})>d_{o} \end{array}\right.$$

In the equation, $U_{rep}$ is the repulsive potential field generated by the obstacle acting on the USV, $k_{r}$ is the coefficient of the repulsive potential field, $X_{o}$ is the position of the obstacle point, $P\;(X,X_{o})$ is the Euclidean distance from the USV to the obstacle point, and $d_{o}$ is the range within which the obstacle exerts its repulsive force on the USV. The repulsive force $F_{rep}$ experienced by the USV is derived from the negative gradient of the repulsive potential field, and the formula is

(4) $$F_{rep}(X)=\left(\begin{array}{c} k_{r}\left(\frac{1}{P(X,X_{o})}-\left.\frac{1}{d_{o}}\right)\right.\frac{1}{P^{2}(X,X_{o})},0\leq P(X,X_{o})\leq d_{o}\\ 0,P(X,X_{o})>d_{o} \end{array}\right.$$

The resultant force potential field experienced by the unmanned ship is denoted as $U_{total}$, and the formula is

(5) $$U_{total}(X)=U_{att}(X)+U_{rep}(X)$$

**Figure 5:**
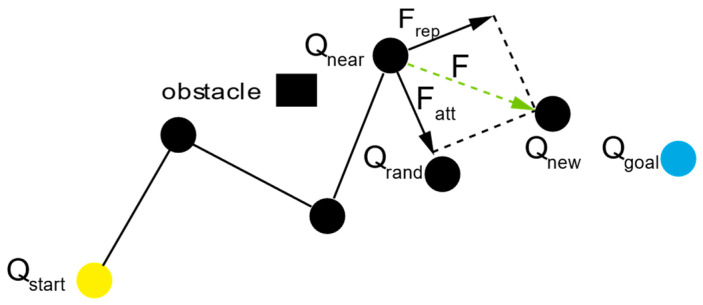
Schematic diagram of the RRT* and APF fusional algorithm.

In the fusion algorithm of the RRT* and the APF algorithm, the starting point is $Q_{near}$, and the target point is $Q_{rand}$. The position of $Q_{new}$ is calculated based on the resultant potential field function. Figure 5 illustrates the process of the RRT* and APF fusional algorithm.

## 4. Algorithm Improvement

### 4.1. Framework of BN-RRT*

When the USV detects that it is trapped, it first determines a target point (with the assumption that reaching this point will ensure escape from the trap). The USV then adds a virtual obstacle in front of the trapped point using a predefined formula. This virtual obstacle is a point calibrated on the map that exerts a repulsive force on the USV. Whenever the USV collides with a real obstacle, a virtual obstacle is added until it reaches the target point.

**Figure 6:**
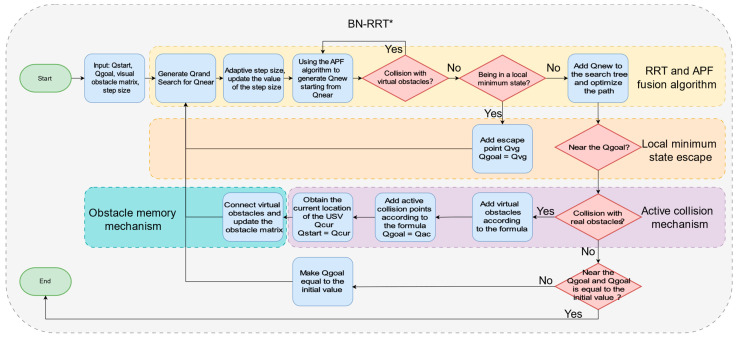
Framework diagram of the BN-RRT* algorithm.

During the process of escaping the predicament, with each collision, the USV’s perception of the trapped environment is further refined. This process is analogous to the principle of blind pathfinding, where a person without sight uses a cane to detect obstacles and ensure the safety of their path. Similarly, USVs without visual or radar sensors rely on an active collision strategy to increase the frequency of collisions and guide the USV to explore in the direction opposite to the water flow, thereby enhancing the efficiency of the rescue operation. Additionally, an obstacle memory mechanism is introduced to connect the added virtual obstacles, reconstructing the trapped terrain environment. This helps prevent the search tree from exploring unfeasible areas and reduces the overall path length. Figure 6 illustrates the framework of the BN-RRT* algorithm.

### 4.2. Virtual Obstacle Addition Mechanism

When the collision sensor carried by the USV detects a collision and the GPS location information does not change within a certain period of time, it can be determined that the USV is trapped. When the USV becomes trapped, a virtual obstacle is generated at the point of impact based on a predefined formula to create a repulsive force. This prevents the USV from colliding with that portion of the obstacle again during the rescue process. The formula for the virtual obstacle mechanism is as follows:

(6) $$\left\{\begin{array}{c} x_{\mathrm{obs}}=x+k_{o}i\\ y_{\mathrm{obs}}=y+k_{o}j \end{array}\right.$$

In the formula, $\left(\left.x_{\mathrm{obs}},y_{\mathrm{obs}}\right)\right.$ is the position coordinate of the virtual obstacle, $\left(\left.x,y\right)\right.$ is the current position coordinate of the USV, $k_{o}$ is a positive-valued distance adjustment parameter, and $\left(i,j\right)$ is the unit vector pointing from the path node before the collision point toward the direction of the collision point. By using this formula, virtual obstacles can be set at the edge of real obstacles without interfering with the subsequent exploration of the USV. This approach prevents the USV from avoiding areas it has already collided with and encourages further exploration into unknown environments.

### 4.3. Active Collision Strategy

The USV perceives the trapped environment by colliding with real obstacles and adding virtual obstacles during the escape process. Each collision represents an increment in the USV’s understanding of the trapped environment, which is similar to the principle of a cane used by a blind person. Therefore, this article proposes an active collision strategy based on practical conditions. During the USV’s escape, when it encounters a real obstacle, an active collision point is added using the ellipse method. This helps the USV perceive the surrounding trapped environment and increases the frequency of collisions.

**Figure 7:**
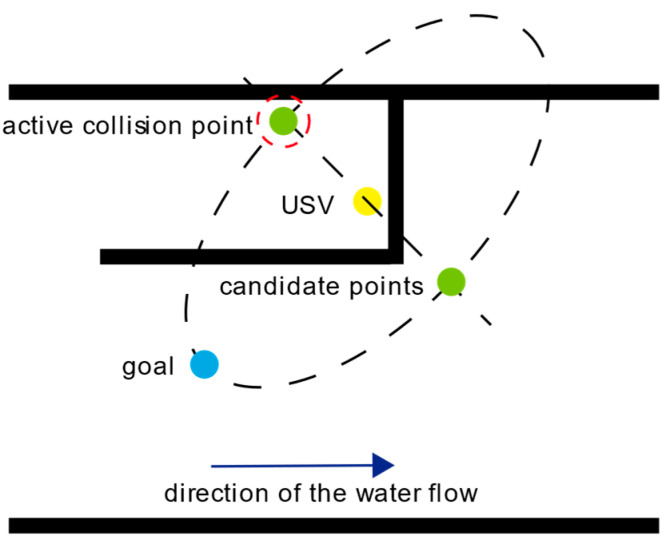
Schematic diagram of the active collision strategy.

The ellipse method defines an ellipse where the Euclidean distance between the current position of the USV and the target point serves as the major axis. The length of the minor axis is set to be equal to that of the major axis. A straight line is drawn perpendicular to the major axis at its midpoint, and the points where this line intersects the ellipse are considered the active collision points and candidate points. The endpoint that lies in the opposite direction of the water flow is selected from the two candidate points, and the active collision point is set as the target point. Choosing candidate active collision points in the direction opposite to the water flow guides the USV to actively explore the surrounding environment of the path taken when trapped, directing it toward the target point. On a USV equipped solely with collision sensors for environmental perception, actively exploring the boundaries of obstacles along the entrance direction of the trapped environment, by retracing the previously traveled path, proves to be more efficient than persistently searching for other exits. This approach demonstrates its versatility and effectiveness across multiple trapped scenarios. Figure 7 provides a schematic diagram of the active collision strategy.

When the USV detects a collision with a real obstacle, the active collision mechanism is triggered. This mechanism constructs an ellipse based on the Euclidean distance between the USV’s current position and the target point. Two candidate points are then determined by the intersections of the ellipse with a vertical line passing through the midpoint of the major axis. The USV selects the candidate point in the direction opposite to the water flow as the active collision point. This point serves as a guide for the USV to explore the surrounding environment in the next step. By following these steps, the USV utilizes the active collision mechanism to incrementally perceive and explore the trapped environment, ultimately achieving its escape.

### 4.4. Local Minimum Escape Strategy

**Figure 8:**
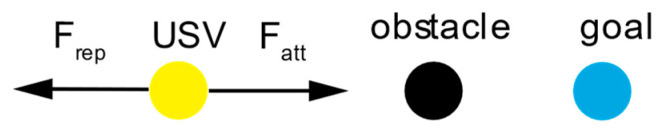
Schematic diagram of the local minimum state.

Due to the introduction of the APF algorithm, when the USV is within the range of repulsive influence, there may be a local minimum problem, which can cause the USV’s path to oscillate or stagnate, leading to a failure to escape. Therefore, improvements are necessary to address this issue. This can be achieved by adding escape points and applying additional gravitational force to the USV to break its current state, thereby enabling it to escape from the local minimum. Figure 8 is a schematic diagram of the local minimum state.

Real-time detection of whether the USV has fallen into a local minimum during the rescue process occurs. Firstly, set a judgment value of d, then obtain the coordinates $\left(x_{t},y_{t}\right)$ of the unmanned underwater platform at the current time t. After a certain time interval, such as t+5, obtain the coordinates $\left(x_{t+5},y_{t+5}\right)$ of the USV again. If the distance between the position coordinates at the two times is less than the judgment value, it can be considered that the unmanned underwater platform has fallen into a local minimum point at this time. The formula for adding escape points is as follows:

(7) $$\left\{\begin{array}{c} x_{ep}=x_{fobs}+\alpha_{1}v+\beta_{1}\\ y_{ep}=y_{fobs}+\alpha_{2}w+\beta_{2} \end{array}\right.$$

In the formula, $\left(x_{ep},y_{ep}\right)$ is the position of the escape point, $\alpha_{1}\alpha_{2}$ is a positive-valued distance adjustment parameter, $\left(x_{fobs},y_{fobs}\right)$ is the position of the virtual obstacle closest to the USV in the selected water flow direction, $\left(v,w\right)$ is the unit vector pointing from the current position of the USV to the virtual obstacle position, and $\beta_{1}\beta_{2}$ is also a distance adjustment parameter with a constant value, which adjusts the position of the escape point in the direction opposite to the water flow. After detecting a local minimum state, the target point is set as the escape point. At this stage, the escape point exerts an attractive force on the USV, effectively guiding it out of the local minimum and facilitating its escape. This formula ensures that the USV successfully escapes from the local minimum state while guiding it to explore unknown areas, thereby improving the efficiency of the escape process.

### 4.5. Obstacle Memory Mechanism

In the application environment of this study, the USV is required to operate in water for extended durations. Therefore, efficiently escaping from trapped conditions is crucial to conserve power. During the escape process, the complex and dynamic nature of real environments complicates the ability to perceive the complete landscape through simple collision detection alone. In contrast, the terrain of constrained environments is often continuous. Based on this observation, we propose an obstacle memory mechanism. This mechanism connects the virtual obstacle points sequentially and treats the intermediate connection lines as no-go exploration zones. These zones are designed to prevent the exploration tree from expanding into areas between obstacles. Importantly, the connection lines do not exert any additional forces on the USV. This operation abstracts the originally complex terrain into straight lines, filtering out some of the intricate details and allowing the USV to bypass non-essential areas while constraining the extension direction of the exploration tree. The inclusion of this mechanism improves the USV’s escape efficiency, reduces the number of iterations required, and lowers the computational load. Moreover, it conserves power, allowing the USV to operate for a longer duration and collect more data in the water.

### 4.6. Adaptive Step Size Mechanism

In the escape algorithm, due to the addition of numerous virtual obstacles after collisions, the distance between the USV and these obstacles becomes very small, leading to situations where the steering angle is excessively large during path planning. In such cases, a fixed step size makes it difficult to smooth the path. Therefore, this study adopts an adaptive step size mechanism. When the distance to the obstacle is too small, the mechanism uses a smaller step size; when the distance to the obstacle allows, a larger step size is employed. The formula is as follows:

(8) $$\left\{\begin{array}{c} L=L_{\max},d_{obs}\geq d_{o}\\ L=L_{\max}\frac{d_{obs}}{d_{o}}+L_{\min}(1-\frac{d_{obs}}{d_{o}}),d_{set}<d_{obs}<d_{o}\\ L=L_{\min},d_{obs}<d_{set}\leq d_{o} \end{array}\right.$$

In this formula, L is the step size, $L_{\max}$ is the maximum step size, $L_{\min}$ is the minimum step size, $d_{obs}$ is the closest distance from the USV to the virtual obstacle, $d_{o}$ is the influence range of the obstacle, and $d_{set}$ is a pre-set value that is smaller than the obstacle’s influence range. When the USV is outside of the obstacle’s influence range, the step size is set to the maximum value; when the distance between the USV and the obstacle is less than the set value, the step size is set to the minimum. Because the paths planned by the algorithm tend to have sharper turns near obstacles, using the minimum step size makes the path more detailed, contributing to path smoothing and enhancing practicality.

### 4.7. Path Smoothing Mechanism

In the application context of this paper, there are two primary reasons for the lack of path smoothness. The first is the large turning angles that occur when the USV steers away from collision points after colliding with real obstacles. The second is the inherent lack of smoothness when the RRT* algorithm connects nodes. Although the proposed algorithm incorporates an Adaptive Step Size Mechanism, the resulting paths may still fail to align with the USV’s mathematical model. To address these issues, a combination of sliding average and steering angle limitation methods is employed for path smoothing, ensuring that the paths planned by the BN-RRT* algorithm are smooth and conform to the requirements of the USV’s mathematical model.

**Figure 9:**
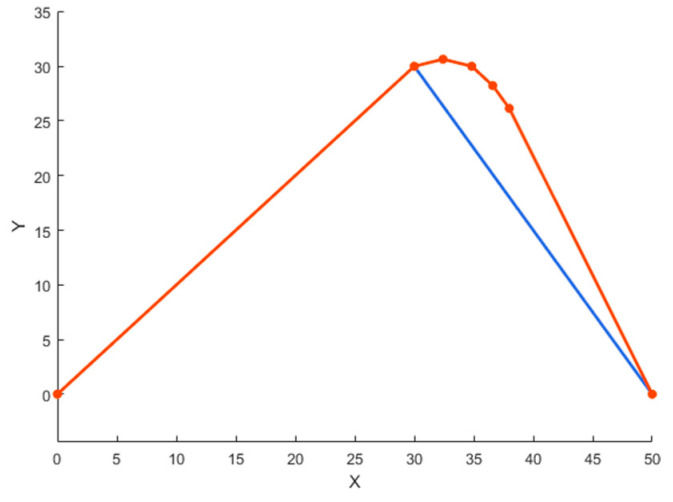
Schematic diagram of an angle limitation method.

To address the first issue, the steering angle limitation method is employed. Initially, a steering angle threshold $\theta_{set}$ is set, and each segment of the path is checked sequentially to determine whether the turning angle θ exceeds this threshold. When θ exceeds $\theta_{set}$, multiple smoothing points are inserted into the path after the collision without altering the path before the collision, gradually changing the direction of the path until θ becomes less than $\theta_{set}$. This method effectively reduces sharp turns in the path, making it more suitable for USV movement. Figure 9 is a schematic diagram of the angle limitation method. The orange lines depict the path after angle limitation, the blue lines show the original path, and the orange dots represent the smoothing points added to the path.

To address the second issue, the sliding average method is employed. The paths generated by the RRT* algorithm are formed by connecting nodes without considering the smoothness of the path, thus requiring additional algorithms for path smoothing. The sliding average method smooths the path by calculating the average value of several points before and after each path point. Specifically, given a set of data points $x_{1},x_{2},\ldots ,x_{n}$, for the $i^{th}$ data point, the sliding average value $y_{i}$ is the average of the point itself and its surrounding m points. The formula is as follows:

(9) $$y_{i}=\frac{1}{m}\sum_{j=i-\frac{m}{2}}^{i+\frac{m}{2}}x_{j}$$

## 5. Algorithm Simulation and Analysis

This section will experimentally verify the BN-RRT* algorithm and compare it with existing RRT, RRT*, and AFF-RRT* algorithms in the same simulated trapped environment to evaluate the performance of the BN-RRT* algorithm in path planning for escape scenarios. The simulation platform is MATLAB R2023b on a 64-bit Windows 11 system configured with an i7-12700 processor running at 2.1 GHz and equipped with 32 GB of RAM.

The experimental parameters include a maximum step size of 20 and a minimum step size of 3. The RRT* algorithm optimizes the path within a range twice the maximum step size. The impact range of obstacles is 150, the maximum number of iterations for a single pathfinding attempt is 10,000, and the maximum number of virtual obstacles is 50. The map size for all experimental stages is [1000 × 1000], with starting and ending points of [480, 500] and [900, 450], respectively. The maximum steering angle $\theta_{set}$ is set to 30 degrees, and the distance $d_{set}$ between the USV and the obstacle is set to 40. The USV is simplified as a particle.

### 5.1. Simulation of Wall-Type Obstacle Escape

Wall-type obstacles generally appear on both sides of a watershed. Over time, continuous water flow erodes and forms these structures. The primary difficulty in escape operations within such environments lies in the continuous, large-scale obstacles blocking the path to the target point. Given only collision detection and USV position information, this terrain holds significant value for simulation.

**Figure 10:**
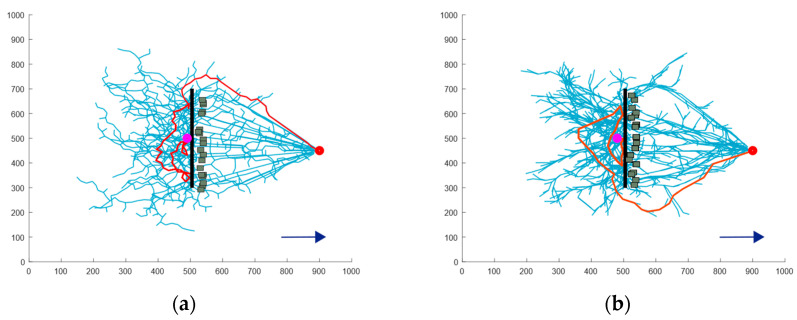

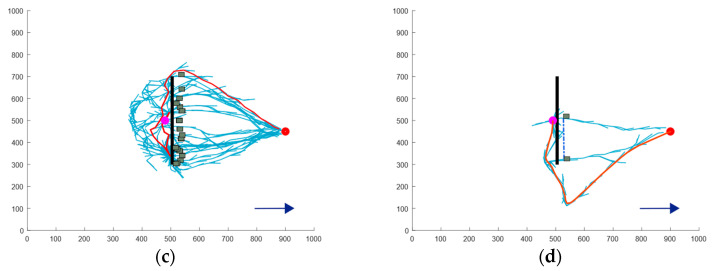
Comparison of escape paths for wall-type obstacle: (**a**) the RRT algorithm; (**b**) the RRT* algorithm; (**c**) the APF-RRT* algorithm; (**d**) the BN-RRT* algorithm.

In Figure 10, the pink dot represents the starting point, the red dot represents the target point, the red line represents the final path, the blue line indicates the sampling path expanded by the search tree, the thick black line denotes real obstacles, the small gray squares represent virtual obstacles generated by the algorithm, and the blue arrow indicates the direction of the water flow. By comparing the four planning results shown in the figure, it is evident that the BN-RRT* algorithm is more concise and effective. The addition of an active collision mechanism enables the USV to actively explore in the direction opposite to the water flow during the rescue process.

Table 1 presents the simulation data for wall obstacles obtained from different algorithms, with each datum calculated from 100 experimental trials. The data demonstrate that the BN-RRT* algorithm shows certain improvements over other algorithms in various metrics.

Analysis of Table 1 indicates that, compared to the APF-RRT* algorithm, the path length planned by the BN-RRT* algorithm has decreased by 10.47%. Additionally, the average running time has decreased by 67.27% compared to the RRT algorithm. These results suggest that the BN-RRT* algorithm demonstrates good performance in scenarios involving wall-type obstacles.

### 5.2. Simulation of Concave Obstacle Escape

**Figure 11:**
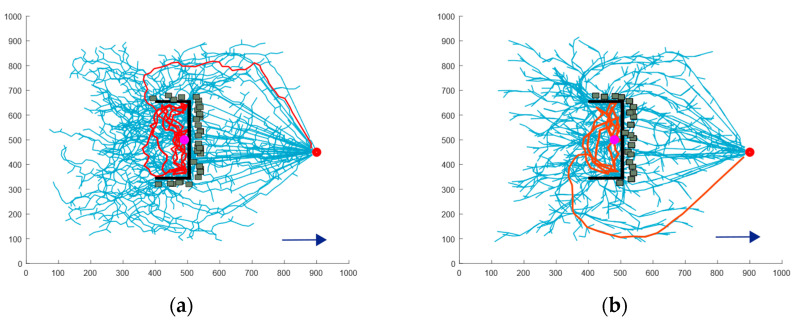

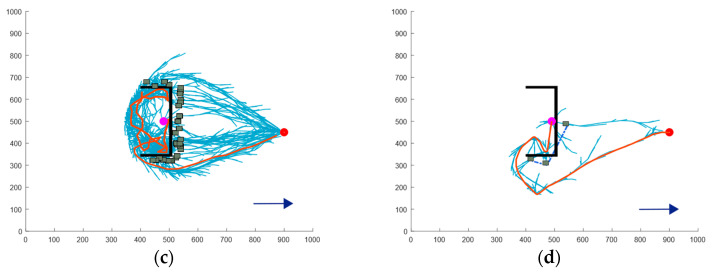
Comparison of escape paths for concave obstacle: (**a**) the RRT algorithm; (**b**) the RRT* algorithm; (**c**) the APF-RRT* algorithm; (**d**) the BN-RRT* algorithm.

Concave obstacles commonly appear at the central islands of watersheds, and the primary challenge in these environments lies in navigating around the concave corners. Because concave obstacles represent a typical challenge for escape strategies in traditional RRT algorithms, this type of obstacle is selected for evaluation. Figure 11 illustrates the escape paths for concave obstacles generated using four algorithms. Correspondingly, Table 2 provides the simulation data comparing these algorithms in concave obstacle scenarios.

As shown in Figure 11a,b, the blue exploration trees extend across the center and periphery of the concave obstacles. This occurs because the RRT and RRT* algorithms are sampling-based methods. When the USV is positioned at the center of a concave obstacle, these algorithms expend substantial time and computational resources expanding the search tree toward randomly sampled points. Consequently, they exhibit low search efficiency and generate suboptimal path quality. However, the RRT* algorithm benefits from an added path optimization mechanism, resulting in a 13% improvement in average path length and a 38.70% reduction in the average number of nodes compared to the RRT algorithm.

The APF-RRT* algorithm, which incorporates the APF algorithm, introduces greater directionality in search tree expansion. Nevertheless, in the absence of sensor information about obstacles, this enhanced directionality slows down the planning process. As a result, the average runtime of the APF-RRT* algorithm increases by 1.33 s compared to the RRT* algorithm.

In contrast, the BN-RRT* algorithm proposed in this paper incorporates an active collision mechanism, enabling a more directed exploration process. It favors exploration in directions opposite to the water flow, which proves particularly efficient in environments where information about trapped conditions is limited. Analysis of the data reveals that the BN-RRT* algorithm improves the average path length and the average number of nodes by 48.50% and 37.87%, respectively, compared to the RRT* algorithm, which otherwise exhibited the best planning effect. Additionally, the BN-RRT* algorithm’s average running time was decreased by 69.14% compared to the RRT algorithm.

### 5.3. Simulation of Deep Concave Obstacle Escape

**Figure 12:**
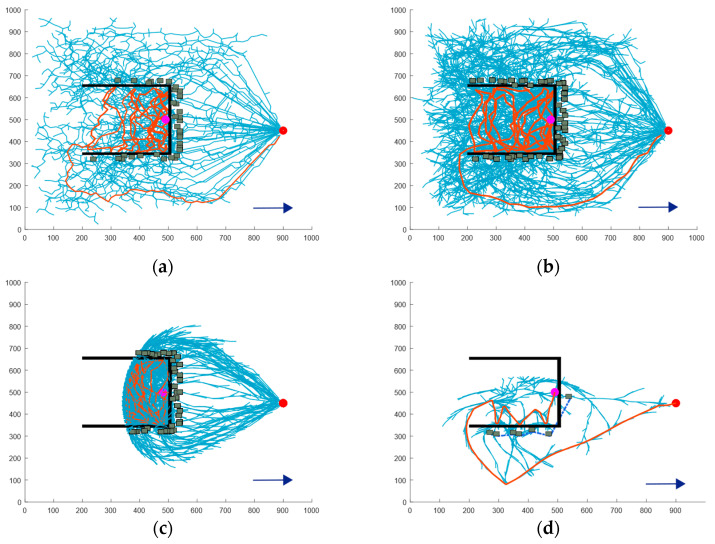
Comparison of escape paths for deep concave obstacle: (**a**) the RRT algorithm; (**b**) the RRT* algorithm; (**c**) the APF-RRT* algorithm; (**d**) the BN-RRT* algorithm.

Due to erosion caused by water flow in the natural environment, the depth of concave obstacles tends to increase over time. Therefore, a deep concave obstacle is included in the simulation to model scenarios in which a USV may become trapped, thereby enhancing the realism of the scenario. Figure 12 illustrates the path planning results for four algorithms. Table 3 presents the simulation data for different algorithms in deep concave obstacle scenarios.

As shown in Figure 12a,b, as the concavity of the obstacles deepens, the success rate of the RRT and RRT* algorithms in escaping from trapped conditions significantly declines. However, the inherent randomness of these algorithms still provides a slight possibility for successful escape. On the other hand, the APF-RRT* algorithm struggles in deeply concave obstacle environments due to its strong guidance, which forces it to rely on path enumeration and trial and error to identify an escape route. This method is highly inefficient and often exhausts the iteration limit without achieving escape.

In contrast, the BN-RRT* algorithm proposed in this paper is specifically optimized for scenarios without sensor information. It incorporates an active collision mechanism inspired by the principle of a white cane. After each collision, an active collision point is added, guiding the USV to explore unknown directions and increasing the frequency of collisions. Additionally, an obstacle memory strategy is introduced, connecting the virtual obstacles created after each collision to prevent the USV from exploring unnecessary areas.

As illustrated in Figure 12c, the APF-RRT* algorithm repeatedly explores the same incorrect directions during its trial and error process, leading to wasted time. The obstacle memory strategy incorporated into the BN-RRT* algorithm effectively addresses this issue. Additionally, this mechanism prevents the USV from exploring the central regions of multiple virtual obstacles. In such scenario, the repulsive forces exerted by multiple obstacles may hinder the USV’s ability to escape. Therefore, the BN-RRT* algorithm achieves a higher success rate compared to other algorithms.

Data analysis reveals that the average path length and planning time of the proposed algorithm were reduced by 64.68% and 70.89%, respectively, compared to the RRT algorithm, which demonstrated the best planning effect. Additionally, the average number of nodes increased by 57.71% compared to the RRT* algorithm.

**Figure 13:**
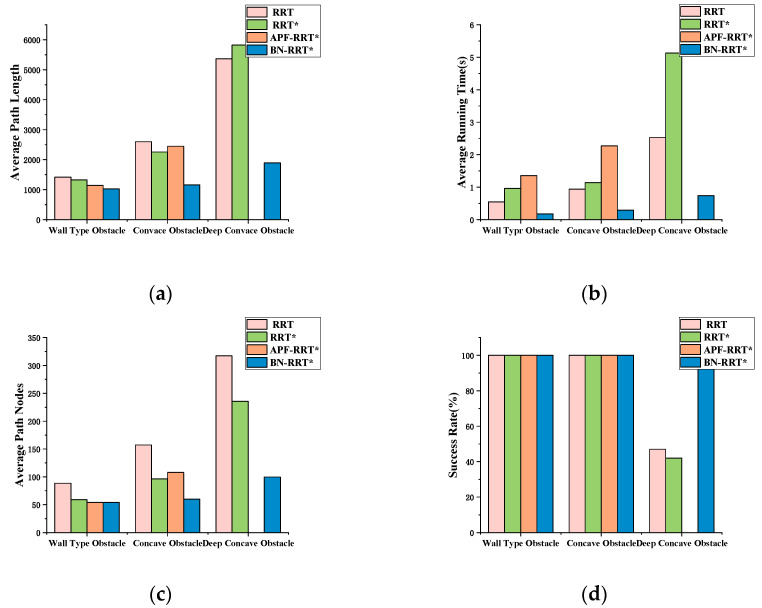
Simulation data comparison graph in three environments: (**a**) average path length; (**b**) average running time; (**c**) average path nodes; (**d**) success rate.

The average path length, running time, average number of path nodes, and success rate generated by each algorithm in three different environments are compared and presented in Figure 13. Each algorithm was run 100 times, and the average path length was recorded. A bar chart, as shown in Figure 13a, illustrates that the BN-RRT* algorithm generates the shortest path in all environments. Figure 13b–d depict the average running time, the average number of nodes, and success rate data, respectively. It can be seen that the algorithm proposed in this paper demonstrates significant improvements over traditional algorithms, particularly in deep concave environments.

### 5.4. Simulation of Obstacle Memory Mechanism

**Figure 14:**
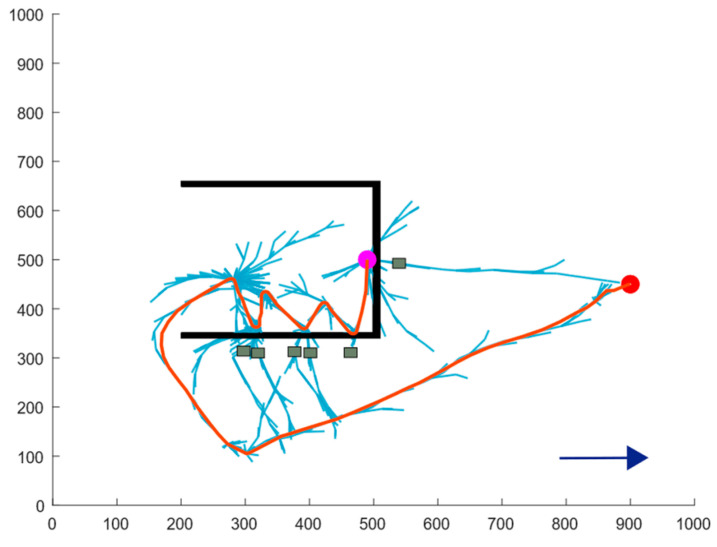
Path planning without obstacle memory mechanism in deep concave obstacle.

Figure 14 illustrates the simulation of the BN-RRT* algorithm without the obstacle memory mechanism when navigating deep concave obstacles. Compared with Figure 12d, it is evident that the algorithm without the obstacle memory mechanism exhibits a significantly denser exploration tree beneath the obstacle. This is because the virtual obstacle setting consists of discrete points with no connections between them, allowing the exploration tree to penetrate the gaps. When the target point acts as an active collision point, the exploration tree extends toward it from both sides of the obstacle. However, the exploration tree extending in the direction of the water flow may cause the USV to move in the wrong direction during the escape process. Therefore, the obstacle memory mechanism is employed to connect virtual obstacles and prevent the exploration tree from sampling in incorrect directions.

Table 4 presents a comparison of the effectiveness of the algorithms with and without the obstacle connection mechanism. An analysis of 100 runs shows that the BN-RRT* algorithm, when incorporating the obstacle memory mechanism, improves the average path length, the average running time, and the average number of path nodes by 13.34%, 6.73%, and 15.89%, respectively, compared to the algorithm without the obstacle memory mechanism. There is no significant difference in the success rate between the two, indicating that the obstacle memory mechanism helps USVs escape from unknown trapped environments.

## 6. Conclusions

This article proposes a BN-RRT* algorithm based on the RRT* algorithm to address the issue of USVs becoming easily trapped in water environments during practical applications. This algorithm utilizes the positioning information provided by the GPS onboard the USV and combines collision detection data from collision sensors to navigate out of trapped spaces. Compared to the traditional RRT* algorithm, the proposed algorithm introduces the following improvements. (1) The integration of the APF algorithm reduces the randomness inherent in the RRT* algorithm. (2) Inspired by the principle of blind pathfinding, an active collision strategy is introduced, which establishes active collision points to help USVs perceive trapped environments and explore in the direction opposite to the water flow, thereby enhancing escape efficiency. (3) An obstacle memory mechanism is proposed to connect virtual obstacles perceived during collisions, preventing USVs from exploring in the wrong direction during the escape process.

The simulation results demonstrate that in the most complex deep concave obstacle environment, the BN-RRT* algorithm reduces the average path length and planning time by 64.68% and 70.89%, respectively, compared to the RRT algorithm. Compared to the RRT algorithm, the average number of nodes decreased by 68.56%. Additionally, the obstacle memory mechanism proposed in this study improved the average path length, planning time, and number of nodes by 13.34%, 6.73%, and 15.89%, respectively, compared to the algorithm without the obstacle memory mechanism. These improvements successfully optimize the algorithm with the inclusion of the BN-RRT* algorithm.

The algorithm is specifically designed for low-cost autonomous drifting USVs used for acquiring bathymetric data in large water bodies. It not only reduces the overall cost of USVs but also ensures their efficient escape capability when entrapped. The research team plans to further explore the potential applications of the BN-RRT* algorithm with the aim of adapting it to diverse aquatic environments. Additionally, future work will focus on integrating more sensors into the USVs and conducting escape experiments to identify the most cost-effective solution for underwater terrain data acquisition.

## Figures and Tables

**Table 1:** Algorithm comparison in wall-type obstacle.

Algorithm	Average Path Length	Average Running Time (s)	Average Path Nodes	Success Rate (%)
RRT	1417.34	0.55	88.39	100
RRT*	1327.16	0.96	59.28	100
APF-RRT*	1143.72	1.36	54.03	100
BN-RRT*	1023.92	0.18	54.28	100

**Table 2:** Algorithm comparison in concave obstacle.

Algorithm	Average Path Length	Average Running Time (s)	Average Path Nodes	Success Rate (%)
RRT	2597.42	0.94	157.32	100
RRT*	2257.36	1.14	96.38	100
APF-RRT*	2450.52	2.27	108.11	100
BN-RRT*	1161.90	0.29	59.88	100

**Table 3:** Algorithm comparison in deep concave obstacle.

Algorithm	Average Path Length	Average Running Time (s)	Average Path Nodes	Success Rate (%)
RRT	5362.72	2.5281	317.25	47
RRT*	5819.42	5.126	235.82	42
APF-RRT*	\	\	\	\
BN-RRT*	1893.99	0.7359	99.74	93

**Table 4:** Comparison of obstacle memory mechanism algorithms.

Algorithm	Average Path Length	Average Running Time (s)	Average Path Nodes	Success Rate (%)
Comparison Algorithm	2185.57	0.7890	118.58	94
BN-RRT*	1893.99	0.7359	99.74	93

## Data Availability

Data are contained within the article. Further inquiries can be directed to the corresponding authors.
